# Screening for Parkinson's disease with response time batteries: A pilot study

**DOI:** 10.1186/1472-6947-4-14

**Published:** 2004-09-13

**Authors:** Andrew M Johnson, Philip A Vernon, Quincy J Almeida, Linda L Grantier, Rene Singarayer, Mandar S Jog

**Affiliations:** 1 Faculty of Health Sciences, University of Western Ontario, London, ON, Canada; 2 Department of Psychology, University of Western Ontario, London, ON, Canada; 3 Department of Kinesiology, Wilfrid Laurier University, Waterloo, ON, Canada; 4 Department of Clinical Neurological Sciences, London Health Sciences Centre, University Campus, London, ON, Canada

## Abstract

**Background:**

Although significant response time deficits (both reaction time and movement time) have been identified in numerous studies of patients with Parkinson's disease (PD), few attempts have been made to evaluate the use of these measures in screening for PD.

**Methods:**

Receiver operator characteristic curves were used to identify cutoff scores for a unit-weighted composite of two choice response tasks in a sample of 40 patients and 40 healthy participants. These scores were then cross-validated in an independent sample of 20 patients and 20 healthy participants.

**Results:**

The unit-weighted movement time composite demonstrated high sensitivity (90%) and specificity (90%) in the identification of PD. Movement time was also significantly correlated (r = 0.59, p < 0.025) with the motor score of the Unified Parkinson's Disease Rating Scale (UPDRS).

**Conclusions:**

Measures of chronometric speed, assessed without the use of biomechanically complex movements, have a potential role in screening for PD. Furthermore, the significant correlation between movement time and UPDRS motor score suggests that movement time may be useful in the quantification of PD severity.

## Background

The success of dopaminergic interventions in the treatment of Parkinson's disease (PD) symptoms has been significant. Nevertheless, a misdiagnosis of PD can cause psychological trauma and unnecessarily expose patients to PD drugs. Additionally, as new, and possibly neuroprotective, drugs become available for the treatment of PD, early and accurate diagnosis will become increasingly important. As the diagnosis of PD is usually based on subjective clinical assessment of overt symptomatology [1], the need for an objective and reproducible battery of diagnostic tests is great. Medical imagery offers some hope for the objective diagnosis of PD (e.g. ^18^F-dopa positron emission tomography [2]), but these techniques tend to be expensive, and inaccessible to patients living in remote areas. What is truly needed is a low-cost objective test battery that might be used in situations where (a) movement disorder specialists are unavailable to render expert diagnoses, and (b) medical imaging is inaccessible.

Montgomery et al. [3,4] have published one of the better known objective PD batteries, incorporating measures of motor performance, olfaction, and mood. The aggregate of all subtests of this battery has demonstrated good diagnostic properties, with a sensitivity of approximately 70%, and a specificity of approximately 90%. Given that the primary symptoms of PD are motoric [1], however, it is interesting to note that the sensitivity of the motor task in this PD battery is approximately 50% [3,4], indicating that diagnoses based solely on this subtest are not much better than chance. As the predictive power of a battery increases with the addition of each valid and independent subtest, it is important to evaluate motor performance paradigms that may produce better predictive validity.

The global slowing that is consistently demonstrated by PD patients suggests that measures of cognitive or motor speed are logical methods for obtaining quantitative measurements of PD severity. As reaction time (RT) and movement time (MT) have repeatedly been demonstrated to show substantive and significant deficits in PD populations (for a review of this literature, see Gauntlett-Gilbert et al. [5]), these indicators are (by definition) capable of distinguishing individuals with PD from healthy participants. Despite this fact, however, the motor subtest of the PD battery described by Montgomery et al. [3,4] remains the only significant attempt at evaluating diagnostic accuracy with these chronometric indicators. Although this subtest measures both RT and MT, the task is performed in a biomechanically complicated fashion that requires the participant to move his/her hand in an arc (i.e. wrist flexion and extension) to aim at LED targets. This test assesses both rigidity and bradykinesia within the same task – and while this is a conceptually defensible measurement decision, the resulting inter-subject variability may overwhelm group differences, and confound diagnostic accuracy. It is, therefore, worth examining the extent to which a simpler paradigm might be used to distinguish PD patients from healthy participants.

The goal of this study, therefore, is to evaluate the diagnostic properties of a choice reaction time task that uses a simple external response console (i.e. a "button box"), similar to other similar response time tasks extant within the PD literature.

## Methods

**Figure 1:**
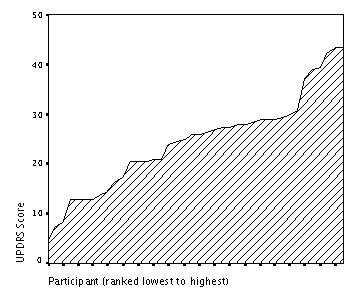
Area graph depicting range of motor impairment in the norming sample (40 patients, 40 controls).

**Figure 2:**
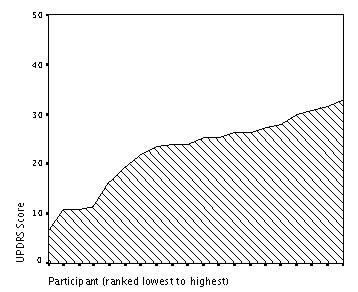
Area graph depicting the range of motor impairment in the cross-validation sample (20 patients, 20 controls).

Two independent samples were drawn for this study, the first consisting of 40 PD patients (Age: M = 62.13, SD = 9.59) and 40 healthy participants (Age: M = 65.02, SD = 8.84), and the second consisting of 20 PD patients (Age: M = 64.50, SD = 10.88) and 20 healthy participants (Age: M = 62.65, SD = 12.02). To ensure that no baseline ability differences existed between groups, Wechsler Adult Intelligence Scale (WAIS) full scale IQ estimates were computed for all participants, using the National Adult Reading Test (NART) [6]. No significant age or IQ differences were identified between patients and controls, in either sample. During the course of testing, patients were also assessed by an experienced clinician (using the motor subscale of the Unified Parkinson's Disease Rating Scale; UPDRS), to determine the severity of their motor symptoms [7]. Patients demonstrated mild to moderate motor symptoms in both the first (M = 24.49, SD = 9.79), and the second sample (M = 22.73, SD = 7.66), with no significant severity differences demonstrated between samples. The spectrum of motor severity within the clinical group is graphically depicted with an area graph in Figures 1 and 2, corresponding to the norming sample and the cross-validation sample, respectively. Finally, all participants were demonstrated to have a Mini-Mental Status Examination (MMSE) score of at least 27 at the time of testing.

The response time tasks used in this study started with an instruction to watch a fixation point (asterisk) in the centre of the computer screen, while depressing the home key (measuring 1.905 cm × 1.905 cm) in the centre of the response console. For the 'uncued' task, participants were not given any advance information concerning the location of the upcoming stimulus. For the 'cued' task, an arrow appeared in place of the fixation point (i.e. in the center of the screen) for a period of 2 seconds, immediately following the disappearance of the fixation point, and correctly cued the location of the upcoming stimulus on all trials. The visual stimulus to which the subject responded was presented on the right or left side of the monitor, at a random interval (between 500 and 1500 ms) following the fixation point ('uncued') or the arrow ('cued'). Participants responded to the stimulus by moving the index finger of their dominant hand from the home key to a response key (measuring 1.905 cm × 1.905 cm) placed 3.175 cm to the left or right of the home key, as directed by the stimulus placement on the screen. The time measured between the onset of the visual stimulus and a participant's movement from the home key was defined as reaction time (RT), and the time measured between a participant's lift from the home key and depression of the response key was defined as movement time (MT). Each task consisted of 10 practice trials, and 40 experimental trials. A participant's RT and MT was computed as the unit-weighted average of scores on the 'cued' and 'uncued' choice response time tasks. This testing apparatus is described in further detail by Johnson et al. [8].

All patients involved in the study were asked to remain drug-free overnight, and to delay taking their morning anti-Parkinsonian medications until after the testing. To avoid any confounding effects resulting from different levels of caffeine intake among participants, all participants were asked to have a normal caffeine-free breakfast prior to testing. None of the participants reported any acute physiological conditions that may have precluded them from putting forth their best effort during the testing session. All procedures and materials were approved by the Health Sciences Research Ethics Board at the University of Western Ontario.

## Results

In the first sample (40 patients and 40 healthy participants), separate receiver operator characteristic (ROC) curves were generated for the composite RT and MT scores. The best prediction (i.e. the largest area under the ROC curve) was achieved using the composite MT score. The cutoff score was identified as the point on the curve that maximized sensitivity, with a specificity of at least 70%. This cutoff score was determined to be 230 ms (i.e. individuals with a MT of at least 230 ms were identified as having PD). To control for the possibility that classification success in the first sample was the result of a capitalization on sample-specific variability [9], this classification strategy (i.e. the cutoff score identified from the ROC within the first sample) was cross-validated in the second sample (20 patients and 20 healthy participants). Classification results and diagnostic efficacy variables for both samples are presented in Table 1.

To identify the extent to which response time predicted disease progression, correlations were computed between the UPDRS and the aggregate RT and MT scores. As the samples demonstrated no significant differences on the UPDRS, correlations were computed across all data collected in both samples. Both RT (r = 0.23, n.s.) and MT (r = 0.59, p < 0.025) were positively correlated with the UPDRS, suggesting that these response time tasks are good predictors of the severity of Parkinsonian symptoms, particularly when considering the MT component of response time.

## Discussion

This study confirms previous research that has shown significant movement time (MT) differences between PD patients and healthy participants [5]. The results of the present study also suggest that MT composites on biomechanically simple response time tasks demonstrate high cross-validated sensitivity and specificity for 'unmedicated' patients (i.e. patients that have been temporarily withdrawn from their dopaminergic medications) – and that these values may be higher than the demonstrated sensitivity and specificity of the motor subtest employed by Montgomery et al. [3,4].

Standardized objective test batteries will be diagnostically useful in two general scenarios: (a) as an adjunct to the physical examination performed by a specialist (to improve diagnostic accuracy), and (b) as a standardized preliminary screening tool, for situations in which a movement disorders expert is unavailable for the physical examination. The latter situation is more important than the former, as primary care physicians are often the first point of contact for these patients. Given the waiting times to see a movement disorders specialist, patients that are considered likely to have PD (based on a screening measure) might be assigned a higher priority in their wait for an initial appointment. This assumes, of course, that primary care physicians will have access to the appropriate motor performance testing devices – and while this is currently prohibitive from a logistical standpoint, it is technologically feasible for the simple tasks described herein to be packaged in smaller (perhaps handheld), less expensive devices.

A MT battery may also allow for the communication of results in a "common metric" – without relying on subjective clinical judgments, thereby complementing other clinical tools. Aside from its diagnostic utility, MT batteries may also be useful in tracking a patient's progress as he/she undergoes treatment. At present, motor evaluations conducted during the clinical exam are the only method for tracking change, and this is considerably more qualitative than the MT measures described herein. Along similar lines, MT batteries could make useful adjuncts to clinical drug trial protocols, as they provide good quantitative measures of motor skill that may be used to gauge the effectiveness of the medication under study – in the present study, MT was able to explain 34.81% of the variability in UPDRS motor scores.

It should, of course, be noted that the present research was only used to separate PD patients from healthy participants, and so it has not been demonstrated to have any differential diagnostic capabilities (e.g. distinguishing PD from progressive supranuclear palsy). Future research in this area should, therefore. investigate the differential diagnostic power of response time batteries – it may be that the MT battery administered in this study are detecting a general 'impairment' factor, and is not useful as a standalone instrument for the diagnosis of Parkinson's disease. Extending the study base to include patients with disorders such as progressive supranuclear palsy would provide important information concerning appropriate norming that may be done to maximize diagnostic utility.

At the very least, however, these results suggest that the use of simple response time batteries may serve as a useful adjunct to other clinical assessment batteries, and may also open interesting avenues of exploration into the consideration of the biological underpinnings of reaction time, and its relationship to movement disorders in general.

## Competing interests

None declared.

## Authors' contributions

AJ conceived and designed the study, collected all data in the norming sample, supervised data collection in the cross-validation sample, supervised data analysis, and contributed to the writing of the paper. PV supervised data collection in the norming sample, assisted in the development of the response time tasks, contributed to the data analysis, and to the writing of the paper. QA assisted with data collection in the cross-validation sample, and contributed to the writing of the paper. LG did all clinical testing on patients in the norming sample. RS assisted with data collection in the cross-validation sample. MJ provided patient diagnoses for participants in the norming sample and the cross-validation sample, and contributed to the writing of the paper.

All authors have read and approved the final manuscript.

## Pre-publication history

The pre-publication history for this paper can be accessed here:



## Figures and Tables

**Table 1:** Classification results

Sample #1 (40 Patients, 40 Controls)				Sample #2 (20 Patients, 20 Controls)			
		Dx by Neurologist				Dx by Neurologist	
					
		PD	Control			PD	Control

Predicted Dx	PD	33	12	Predicted Dx	PD	18	2
			
	Control	7	28		Control	2	18

Sensitivity		82.5%		Sensitivity		90.0%	
Specificity		70.0%		Specificity		90.0%	
